# Complete genome analysis of echovirus 30 strains isolated from hand-foot-and-mouth disease in Yunnan province, China

**DOI:** 10.1186/s12985-023-02179-9

**Published:** 2023-09-20

**Authors:** Ming Zhang, Daqian He, Yuhan Liu, Yue Gong, Wenxun Dong, Ying Chen, Shaohui Ma

**Affiliations:** 1 Institute of Medical Biology, Chinese Academy of Medical Sciences, Peking Union Medical College, Kunming, 650118 PR China; 2 Yunnan Key Laboratory of Vaccine Research Development on Severe Infectious Disease, Kunming, 650118 PR China; 3 Department of Thoracic Surgery I, The Third Affiliated Hospital of Kunming Medical University Yunnan Cancer Hospital,Yunnan Cancer Center), Kunming, 650118 China

**Keywords:** Echovirus 30, Complete genome, Hand-foot-and-mouth disease, Recombination, Selection pressure

## Abstract

**Background:**

Echovirus 30 is prone to cause hand-foot-and-mouth disease in infants and children. However, molecular epidemiologic information on the spread of E30 in southwestern China remains limited. In this study, we determined and analyzed the whole genomic sequences of E30 strains isolated from the stools of patients with hand-foot-and-mouth disease in Yunnan Province, China, in 2019.

**Methods:**

E30 isolates were obtained from fecal samples of HFMD patients. The whole genomes were sequenced by segmented PCR and analyzed for phylogeny, mutation and recombination. MEGA and DNAStar were used to align the present isolates with the reference strains. The VP1 sequence of the isolates were analyzed for selection pressure using datamonkey server.

**Results:**

The complete genome sequences of four E30 were obtained from this virus isolation. Significant homologous recombination signals in the P2-3’UTR region were found in all four isolates with other serotypes. Phylogenetic analysis showed that the four E30 isolates belonged to lineage H. Comparison of the VP1 sequences of these four isolates with other E30 reference strains using three selection pressure analysis models FUBAR, FEL, and MEME, revealed a positive selection site at 133rd position.

**Conclusions:**

This study extends the whole genome sequence of E30 in GenBank, in which mutations and recombinations have driven the evolution of E30 and further improved and enriched the genetic characteristics of E30, providing fundamental data for the prevention and control of diseases caused by E30. Furthermore, we demonstrated the value of continuous and extensive surveillance of enterovirus serotypes other than the major HFMD-causing viruses.

**Supplementary Information:**

The online version contains supplementary material available at 10.1186/s12985-023-02179-9.

## Introduction

Hand-foot-and-mouth disease (HFMD) is a kind of childhood infectious disease. As one of the class C infectious diseases in China, HFMD is common in children under 5 years old, while the main symptoms usually include mouth sores and small blisters on the hands and feet, and may be accompanied by low-grade fever, anorexia, etc. Most cases heal spontaneously in about one week, however, some develop rapidly, leading to severe complications such as aseptic meningoencephalitis, viral myocarditis, etc., and even death. HFMD is caused by more than 20 enteroviruses, among which, enterovirus 71 (EV71) and coxsackievirus A16 (CVA16) are the most common causes [1–3].

Enteroviruses belong to the genus *Enterovirus*, *Picornaviridae*. The main enteroviruses associated with human diseases are EV-A, EV-B, EV-C, and EV-D, of them, Echovirus 30 (E30) belongs to EV-B [2]. In multiple retrospective studies of clinical samples of feces, serum, and cerebrospinal fluid from several countries, E30 infection is the most common causative agent of aseptic meningitis outbreaks in Asia, Europe, and the Americas in recent years [4–7]. In addition, serious infections of E30 have been documented in Zhejiang, Fujian, Jiangsu, and Shandong in China, inflicting an immense burden on families worldwide [8]. In this study, four E30 strains were isolated from fecal samples of children with HFMD, and the whole genome characteristics were analyzed, providing a certain reference for the prevention and control of HFMD.

## Materials and methods

### Sample collection, viral isolation, and adaptive culture

A total of 30 samples were collected from children with symptoms of HFMD in 2019 (Yunnan, China), of those, 4 stool specimens were isolated to obtain E30. The gender ratio was 3:1, with 3 males and 1 female cases. The median age of onset was less than 3.10 years. The specimens were collected during the acute phase of the illness. Human rhabdomyosarcoma (RD) cell, green monkey kidney (Vero) cell, and human embryonic lung diploid fibroblast (KMB-17) cell lines were used for isolation and adaptive culture of E30. After three passages of adaptive culture, samples that induced cytopathic effects (CPEs) were stored at -30 °C.

### Reverse transcription polymerase chain reaction (RT-PCR), sequencing and typing

RT-PCR, sequencing and typing were performed according to a previously described procedure [9]. Briefly, the Body Fluid viral DNA/RNA Miniprep Kit (Axygen, USA). is used to extract viral RNA from the supernatants of infected cells. RT-PCR was performed using the PrimeScript One Step RT-PCR Kit Ver.2 (Takara, Dalian, China).

Primers AN89 and AN88 were used to amplify the part VP1 sequences [10]. The complete genome fragments were amplified and sequenced using multiple pairs of primers, and the amplification and sequencing primers are summarized in Additional file 1(Table S1). The positive amplification products were sequenced by Tsingke Biological Technology Co., Ltd. (Kunming, China). The Enterovirus Genotyping Tool was used for EV classification. The VP1-encoding sequences and complete genomes were compared with sequences available in GenBank using BLAST.

### Selection pressure analysis of the E30 VP1 gene

The selection pressure of E30 VP1 was predicted using datamonkey server [11] (http://www.datamonkey.org) to conduct the selection pressure analysis, four new strains were analyzed by four site selection pressure analysis models: Mixed Effects Model of Evolution (MEME) [12], Fixed Effects Likelihood (FEL) [13], Fast Unconstrained Bayesian App Roximation (FUBAR) [14], Single-Likelihood Ancestor Counting (SLAC) [13]. The *p*-value of MEME, FEL, and SLAC was 0.1, and that of FUBAR was 0.9. Positive selection, neutral selection and negative selection were defined as dN/dS > 1, dN/dS = 1, and dN/dS < 1 respectively, assuming that the selection pressure of each site was constant throughout the phylogeny.

### Phylogenetic analysis and sequence alignment

The Molecular Evolutionary Genetic Analysis (MEGA) version X software with the Neighbor-Joining (NJ) methods with 1000 bootstrap replications was used to perform the phylogenetic analysis of 64 E30 strains (i.e., 60 strains from the GenBank database and 4 strains isolated in this work) and 1 E21 strain based on the complete VP1 sequence (876 nucleotides) [15]. E30 was genotyped according to previous studies [40–42]. The DNAStar software was employed for pairwise alignment of these sequences. The reliability value was evaluated according to the 1000 bootstrap value. Simplot 3.5.1 and RDP4 were used for recombination analysis.

## Results

### Primary characterization and complete genome structure analysis

Two E30 strains 9K3/YN/CHN/2019(9K3), and 15K3/YN/CHN/2019(15K3) were isolated from KMB-17 cells, and two strains 32R3/YN/CHN/2019(32R3) and 52R3/YN/CHN/2019(52R3) were isolated from RD cells. The sequences of 4 E30 strains characterized in this study were deposited in the GenBank database (accession numbers: OQ210941 ~ OQ210944.).

The whole genome sequences of the four strains (9K3, 15K3, 32R3, and 52R3) isolated in Yunnan Province in 2019 were determined. The genome sequences were 7422–7452 nucleotides in length, containing an ORF of 6585 nucleotides, which encoded a polyprotein of 2194 amino acids. The ORF sequence was flanked by a noncoding 5’-UTR of 739–754 nucleotides and a noncoding 3’-UTR of 91–113 nucleotides. The whole-genome nucleotide and amino acid identities of the four isolates were 99.2-99.7% and 98.9-99.5%, respectively. The total base compositions were 28.07–28.23% A, 23.32–23.40% T, 24.50-24.65% G, and 23.89–23.98% C.

The whole genome structure of the four strains was consistent with the structural characteristics of enterovirus. Compared with the E30 prototype Bastianni [16], the four E30 isolates in this study had no base deletion or insertion in the coding region.

### Nucleotide and amino acid homology analysis

**Fig. 1:**
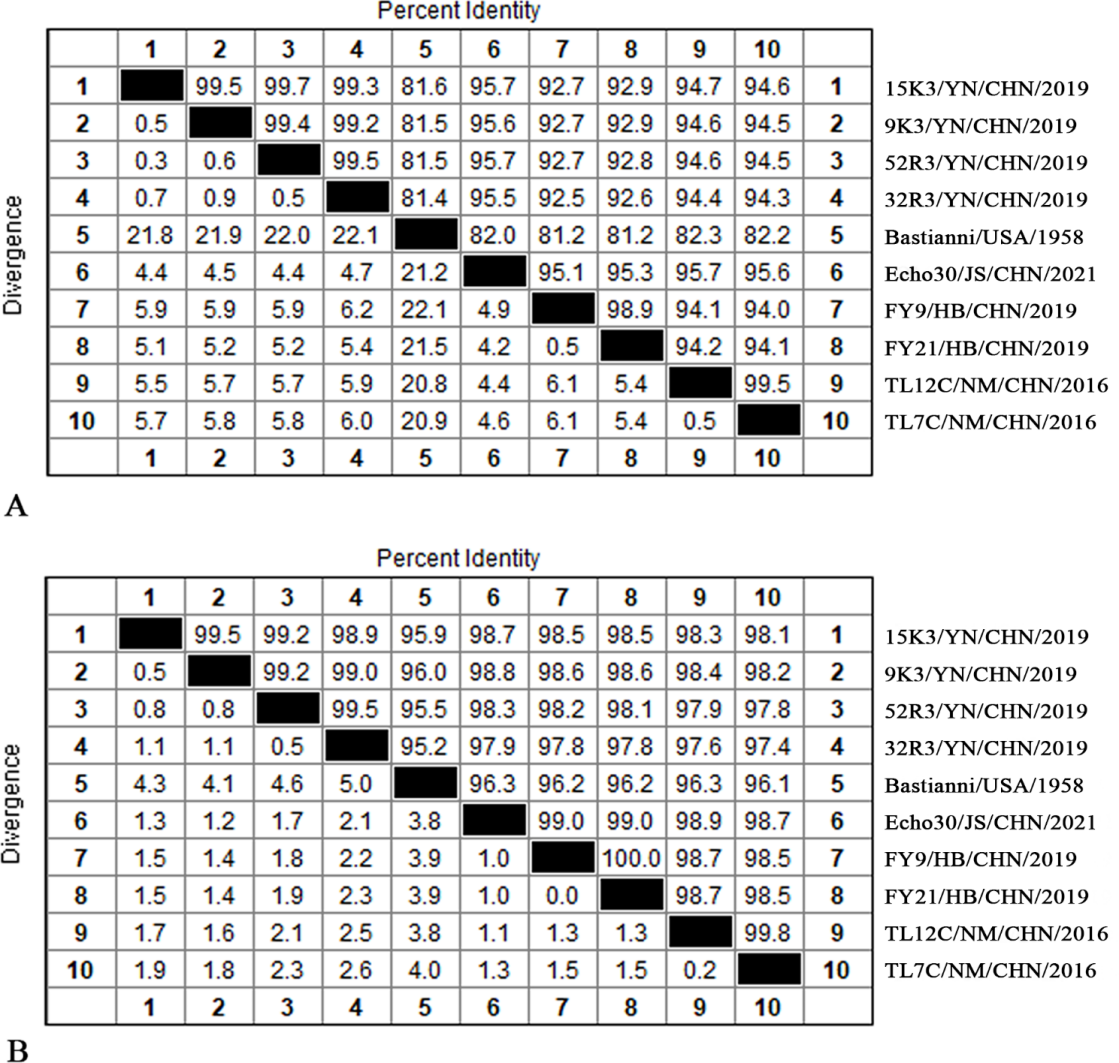
Consistency comparison of nucleotides and amino acids (%). (**A**) nucleotide consistency, (**B**) Amino acid consistency. Homology was shown on the upper right and differentiation was shown on the lower left

Pairwise comparisons of the nucleotide and amino acid sequences of the four strains (9K3, 15K3, 32R3, and 52R3) and the E30 prototype Bastianni strain and other E30 strains are shown in Fig. 1. The results showed that the nucleotide and amino acid sequences percent identities of the four isolates were 99.2-99.7% and 98.9-99.5%, they were 81.4-81.6% and 95.2-96.0% between the four isolates and Bastianni, were 92.5-95.7% and 97.4-98.8% between the four isolates and epidemic strains of China in recent years, respectively.

**Fig. 2:**
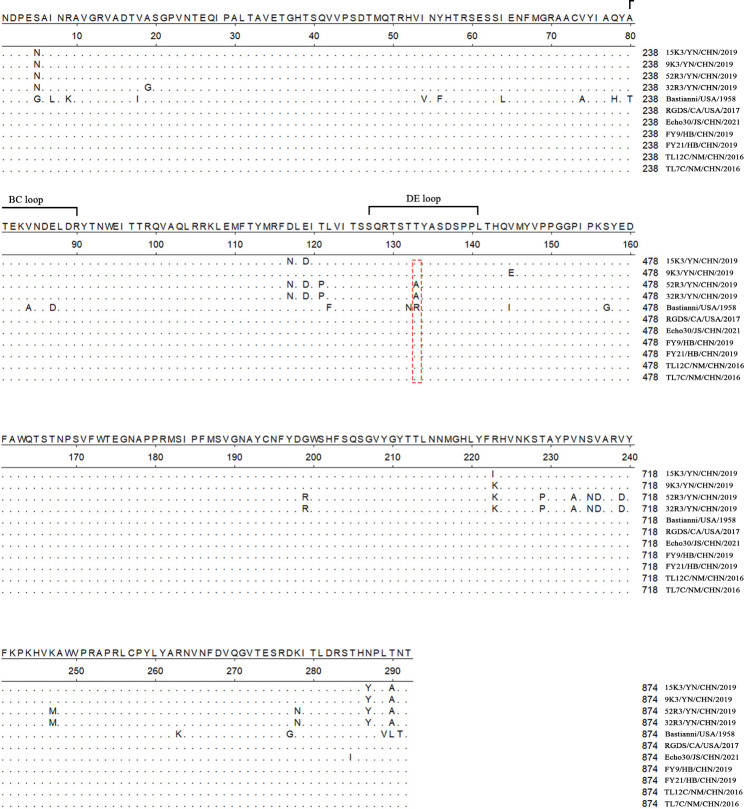
Nucleotide and amino acid mutation sites revealed by the amino acid site mutation analysis of four E30 strains. Hide residues that match the Consensus exactly; Nucleotide sites were listed on the right; Mostly identical amino acids were listed at the top

In the analysis of amino acid site mutation, compared with Bastianni, there were 36 amino acid site mutations in the VP1 protein region in the four new strains (Fig. 2), where T80A, A84V, and D87E were located in the BC loop (80–90) structural region. N132T, R133T, and R133A are located in the structural region DE loop (127–141). Compared with the VP1 protein region of the full-length strains in recent years, there were 18 amino acid site mutations, among which, T133A was located in the structural region DE loop (127–141).

### Phylogenetic analysis

**Fig. 3:**
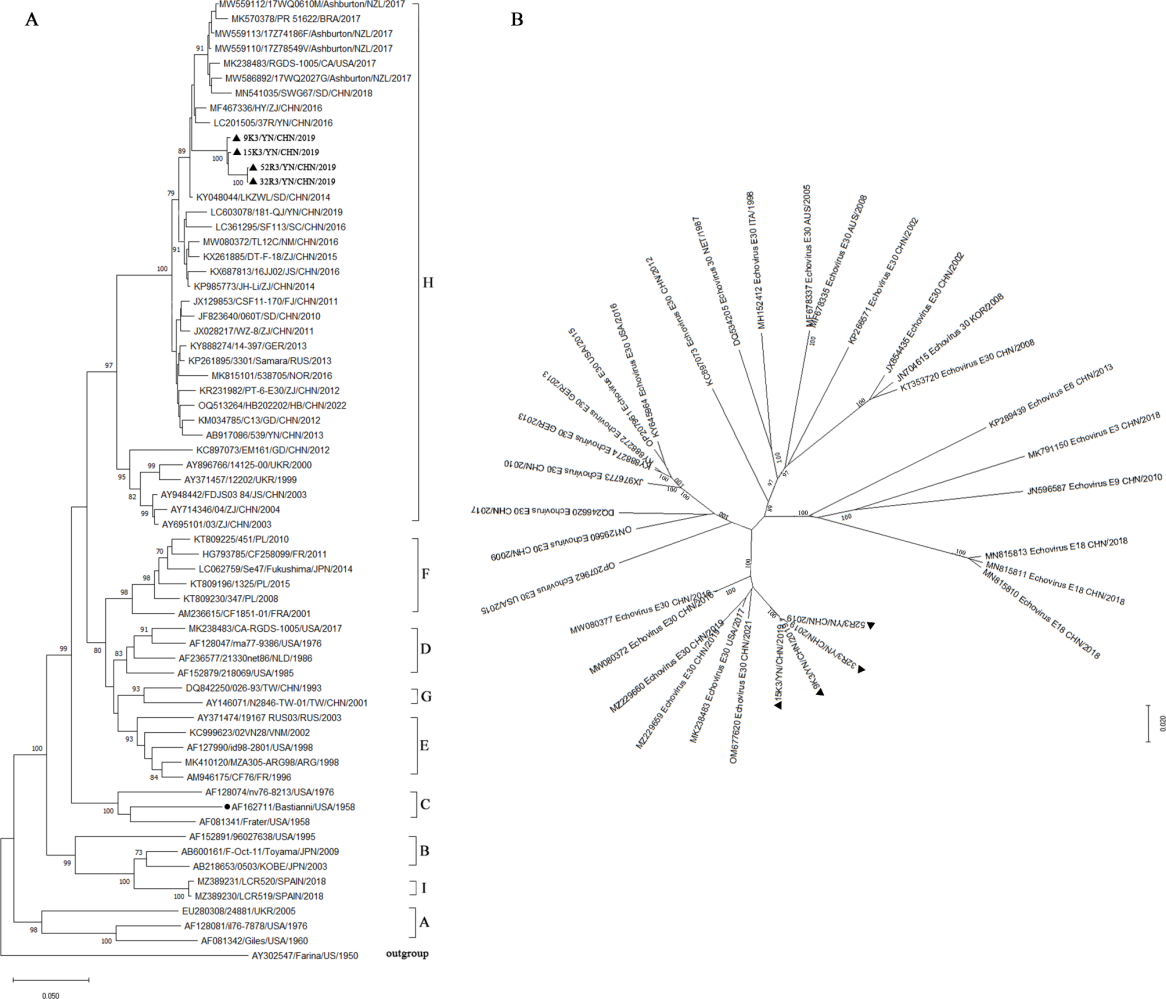
**A** Phylogenetic analysis of E30 based on the VP1 genome. ●. prototype sequences Bastianni (AF162711), ▲. E30 sequences obtained in this study. Neighbor-joining phylogenetic tree (MEGA) after ClustalW alignment of VP1 sequences, Bootstrap resampling (1000 replicates) was used. Values > 70% are shown. echovirus 21 strain Farina (AY302547) was used as an outgroup. **B** Phylogenetic tree based on the complete genome

The 61 whole VP1 sequences available in GenBank were included in an analysis of the four isolates collected in this study. According to the approximate mean 15% cutoff divergence value used to genotype enterovirus [16–18], the E30 strains were divided into eight clusters (A–I). The main epidemic strains belonged to the F and H clusters. The four new strains were all classified in lineage H (Fig. 3).

Cluster H contained most E30 strains (36 strains), including the most Chinese strains, together with isolates from New Zealand, Brazil, the USA, Germany, Russia, Ukraine, and Thailand, collected from 1999 to 2022. Cluster F included seven strains from Poland, France, and Japan, collected in 2001–2015.

The whole VP1 sequences (876 nucleotides) of the four Yunnan strains showed the greatest nucleotide identities (95.3–96.6%) and (94.5–98.6%) amino acid identities with E30 strain 37R/YN/ CHN/2016 (LC201505), isolated from a healthy child in China. The four isolates shared 79.6–80.9% nucleotide identities and 87.7–91.8% amino acid identities with the whole VP1 sequence of the E30 prototype Bastianni strain. The whole VP1 nucleotide and amino acid identities among the four Yunnan isolates were 98.3–99.9% and 95.2–99.7%, respectively.

### Selection pressure analysis

The sites under positive selection pressure were undetected by the SLAC method, and 233 negative selection sites were detected. The number of positive selection sites was 2 detected by the FUBAR method, and the number of negative selection sites was 276. Four new strains were set as foreground branches and the rest as background branches, fifteen positive selection sites were detected using the FEL method. Thirteen positive selection sites were selected by MEME in Additional file 1 (Table S2-S4). FUBAR, FEL, and MEME all detected the 133rd positive selection, as shown in Table 1.

**Table 1:** Positive selection site detected by all three models

Position aa	Method					
	FUBAR		FEL		MEME	
	dN/dS	Post. Pr	dN/dS	*p*-value	ω+	*p*-value
133	3.843	0.938	595.658	0.0018	142.57	0.00

### Recombination analysis

The sequences sharing the high identities of the four strains (9K3, 15K3, 32R3, and 52R3) were screened online with BLAST (Tables 2–5). The 2B、2 C、3A region of the four strains (9K3, 15K3, 32R3, and 52R3) shared high identities (92.93%-94.62%) with that of E6 strain P735/CHN/2013 and E18 strains (S5758/CHN/2018, S6064/CHN/2018 and S6363/CHN/2018) whereas in the 3 C and 3D regions, it shared the high identities (88.34%-94.72%) with the E3 strain 123-R2/CHN/2018, E9 strain KM812/CHN/2010 and E18 strain S6064/CHN/2018. In the 3’-UTR, four strains (9K3, 15K3, 32R3, and 52R3) shared the high identities (94.0-96.77%) with E18 strain S5758/CHN/2018.

**Table 2:** High similarity of nucleotide sequences of enteroviruses in all sequenced genomic regions of the 9K3/YN/CHN/2019 strain

Region	Type	Strain	Nucleotide identity%	Accession number	Disease/isolation source
5’-UTR	E30	CA-RGDS-1005/USA/2017	97.69	MK238483	Meningitis/Cerebrospinal fluid
VP4	E30	1227/RUS/2018	96.67	MT281433	NA*/Cerebrospinal fluid
VP2	E30	CA-RGDS-1048/USA/2017	96.41	MN153801	Meningitis/Cerebrospinal fluid
VP3	E30	WQ2027G/NZ/2017	96.36	MW586892	Aseptic meningitis/Cerebrospinal fluid
VP1	E30	LKZWL/CHN/2014	97.15	KY048044	Aseptic meningitis/Cerebrospinal fluid
2 A	E30	Omsk/RUS/2013	98.77	KU133631	Meningitis/Cerebrospinal fluid
2B	E6	P735/CHN/2013	92.93	KP289439	Hand, foot and mouth disease
2 C	E18	S5758/CHN/2018	94.31	MN815813	NA/Throat swab
3 A	E18	S6363/CHN/2018	94.38	MN815811	NA/Throat swab
3B	E30	CA-RGDS-1048/USA/2017	93.94	MN153801	Meningitis/Cerebrospinal fluid
3 C	E18	S6064/CHN/2018	94.72	MN815810	NA/Throat swab
3D	E3	123-R2/CHN/2018	90.96	MK791150	NA/NA
3’-UTR	E18	S5758/CHN/2018	95.45	MN815813	NA/Throat swab

*NA: Not available.

**Table 3:** High similarity of nucleotide sequences of enteroviruses in all sequenced genomic regions of the 15K3/YN/CHN/2019 strain

Region	Type	Strain	Nucleotide identity%	Accession number	Disease/isolation source
5’-UTR	E30	CA-RGDS-1005/USA/2017	98.75	MK238483	Meningitis/Cerebrospinal fluid
VP4	E30	1227/RUS/2018	97.14	MT281433	NA/Cerebrospinal fluid
VP2	E30	CA-RGDS-1048/USA/2017	96.41	MN153801	Meningitis/Cerebrospinal fluid
VP3	E30	WQ2027G/NZ/2017	96.36	MW586892	Aseptic meningitis/Cerebrospinal fluid
VP1	E30	LKZWL/CHN/2014	97.15	KY048044	Aseptic meningitis/Cerebrospinal fluid
2 A	E30	CA-RGDS-1048/USA/2017	95.78	MN153801	Meningitis/Cerebrospinal fluid
2B	E6	P735/CHN/2013	92.93	KP289439	Hand, foot and mouth disease
2 C	E18	S6363/CHN/2018	94.62	MN815811	NA/Throat swab
3 A	E18	S6363/CHN/2018	94.38	MN815811	NA/Throat swab
3B	E30	CA-RGDS-1048/USA/2017	93.94	MN153801	Meningitis/Cerebrospinal fluid
3 C	E9	KM812/CHN/2010	88.34	JN596587	Hand, foot and mouth disease/NA
3D	E3	123-R2/CHN/2018	90.96	MK791150	NA/NA
3’-UTR	E18	S5758/CHN/2018	96.77	MN815813	NA/Throat swab

**Table 4:** High similarity of nucleotide sequences of enteroviruses in all sequenced genomic regions of the 32R3/YN/CHN/2019 strain

Region	Type	Strain	Nucleotide identity%	Accession number	Disease/isolation source
5’-UTR	E30	CA-RGDS-1005/USA/2017	98.58	MK238483	Meningitis/Cerebrospinal fluid
VP4	E30	1227/RUS/2018	97.14	MT281433	NA/Cerebrospinal fluid
VP2	E30	CA-RGDS-1048/USA/2017	96.41	MN153801	Meningitis/Cerebrospinal fluid
VP3	E30	WQ2027G/NZ/2017	95.38	MW586892	Aseptic meningitis/Cerebrospinal fluid
VP1	E30	LKZWL/CHN/2014	95.89	KY048044	Aseptic meningitis/Cerebrospinal fluid
2 A	E30	Omsk/RUS/2013	98.77	KU133631	Meningitis/Cerebrospinal fluid
2B	E6	P735/CHN/2013	92.93	KP289439	Hand, foot and mouth disease
2 C	E6	P735/CHN/2013	94.13	KP289439	Hand, foot and mouth disease
3 A	E18	S6363/CHN/2018	94.38	MN815811	NA/Throat swab
3B	E30	CA-RGDS-1048/USA/2017	93.94	MN153801	Meningitis/Cerebrospinal fluid
3 C	E9	KM812/CHN/2010	88.34	JN596587	Hand, foot and mouth disease/NA
3D	E3	123-R2/CHN/2018	90.96	MK791150	NA/NA
3’-UTR	E18	S5758/CHN/2018	95.96	MN815813	NA/Throat swab

**Table 5:** High similarity of nucleotide sequences of enteroviruses in all sequenced genomic regions of the 52R3/YN/CHN/2019 strain

Region	Type	Strain	Nucleotide identity%	Accession number	Disease/isolation source
5’-UTR	E30	CA-RGDS-1005/USA/2017	98.75	MK238483	Meningitis/Cerebrospinal fluid
VP4	E30	1227/RUS/2018	97.14	MT281433	NA/Cerebrospinal fluid
VP2	E30	CA-RGDS-1048/USA/2017	96.15	MN153801	Meningitis/Cerebrospinal fluid
VP3	E30	WQ2027G/NZ/2017	96.36	MW586892	Aseptic meningitis/Cerebrospinal fluid
VP1	E30	LKZWL/CHN/2014	96.00	KY048044	Aseptic meningitis/Cerebrospinal fluid
2 A	E30	Omsk/RUS/2013	98.77	KU133631	Meningitis/Cerebrospinal fluid
2B	E6	P735/CHN/2013	92.93	KP289439	Hand, foot and mouth disease
2 C	E18	S6064/CHN/2018	94.62	MN815810	NA/Throat swab
3 A	E18	S6363/CHN/2018	94.38	MN815811	NA/Throat swab
3B	E30	CA-RGDS-1048/USA/2017	93.94	MN153801	Meningitis/Cerebrospinal fluid
3 C	E9	KM812/CHN/2010	88.34	JN596587	Hand, foot and mouth disease/NA
3D	E3	123-R2/CHN/2018	90.96	MK791150	NA/NA
3’-UTR	E18	S5758/CHN/2018	94.00	MN815813	NA/Throat swab

**Fig. 4:**
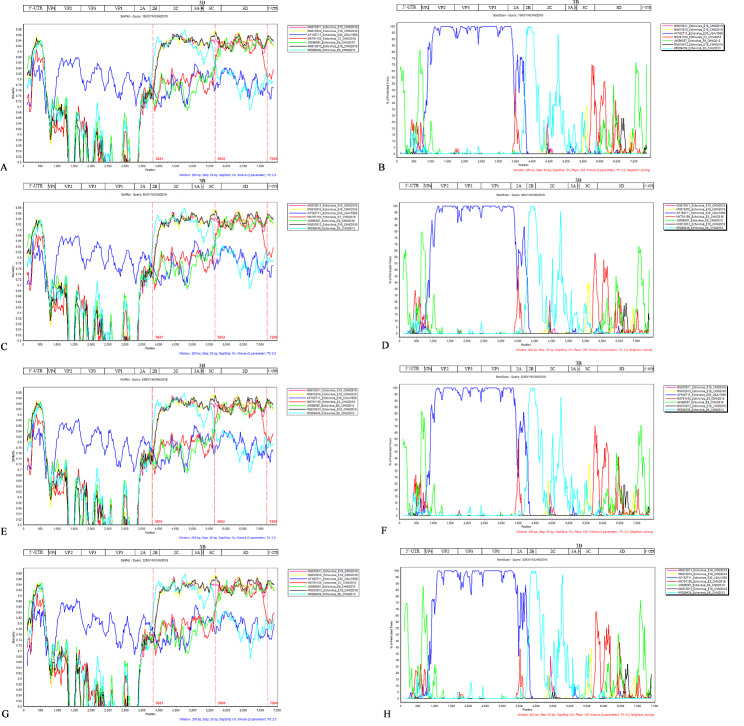
Similarity plot and bootscan analysis of four complete genome sequences compared with other epidemic enterovirus strains. **A**. and **B** analysis of 9K3 strain with closely related strains ; **C** and **D** analysis of 15K3 strain with closely related strains; **E** and **F** analysis of 32R3 strain with closely related strains; **G** and **H** analysis of 52R3 strain with closely related strains. The analyses were conducted via Simplot 3.5.1 with a sliding window of 200 nucleotides moving in steps of 20 nucleotides

Similarity analysis and Bootscan recombination analysis indicated that no recombination occurred in the P1 region of the four Yunnan strains in this study. In the 2B segment of region P2, recombination occurred with other EV-B epidemic strains, and the recombination point was approximately 3800. Moreover, in the 3 C segment of region P3, recombination occurred with other EV-B epidemic strains, and the recombination point was approximately 5600, and in the 3’-UTR, recombination occurred with other EV-B epidemic strains, and the recombination point was approximately 7200 (Fig. 4).

RDP4 analysis was used to reconfirm these recombination events and determine the recombination breakpoint. The recombination was determined by RDP, GENECONV, BootScan, MaxChi, Chimaera, SiScan, and 3Seq methods. The results are consistent with the analysis of Simplot 3.5.1 software in Additional file 1 (Table S5-S6).

## Discussion

HFMD can be caused by a variety of enteroviruses, and EV71 and CVA16 are previously thought to be the major pathogens in Asia [7, 8, 17, 18]. However, with the introduction of the inactivated EV71 vaccine, the proportion of HFMD caused by EV enteroviruses has changed, with other EV serotypes instead of EV71 becoming the predominant etiologic agent [19–21], such as CVA6, CVA10, CVB3, CVB5, etc [1, 2, 9, 22–24]. As E30 is frequently detected in HFMD cases, E30 also has the potential to cause pandemics, suggesting the value of continuous, extensive surveillance of other enterovirus serotypes in addition to the main causative viruses for HFMD [6].

The VP1 coding region of enteroviruses contains the main antigenic neutralization sites and lots of important structural domains. It is used as the basis for molecular typing of enterovirus and plays an important role in the binding and entry process of the virus into the host cells [25–30]. BC and DE loops of the VP1 coding region are important regions which closely related to the antigenicity of the virus, the binding affinity of corresponding specific neutralizing antibodies, and the ability of the virion to enter host cells [31–34]. The phylogenetic tree of complete genome sequences reflects that the four isolates formed a separate evolutionary branch with the 2017 US strain, the 2016 Inner Mongolia, China strain, the 2019 Wuhan, China strain, and the 2021 Jiangsu, China strain, which indicated that domestic and international travel can lead to the exchange of viral strains from different countries and regions and a new epidemic lineage is constructed through recombination, genetic mutation, and other changes together.

Selection pressure analysis revealed that the 133rd loci was detected by all these three methods. The 133rd codon loci of the VP1 sequence is located in the DE loop structural region where equipped with the crucial antigen determinant of virion particles. Compared with prototype strain Bastianni, 15K3 and 9K3 mutated from R to T at the 133rd site, while 52R3 and 32R3 mutated from R to A at the same place. However, 15K3 and 9K3 were isolated from KMB-17 cells, 52R3 and 32R3 were isolated from RD cells, which indicated that E30 evolved with environmental changes, according to the law of adaptive mutation in the process of virus evolution, and this substitution may affect viral adaptation in different cells. In addition, T80A, A84V, and D87E were found in the BC loop, while N132T, R133T, and R133A were found in the DE loop. The mutations may affect the evolution of the virus, but further research is needed.

E30 outbreaks present a periodic pattern of three to five years, lasting one to two years, which are usually associated with the rapid spread of different strains [4, 35]. It has been reported that recombination is common for EV-B, and the non-structural protein (NSP) region is the hot spot of recombination and leads to the emergence of new epidemic strains [36–39].

Recombination is a major driver in the evolution of enteroviruses, especially for EV-B [37]. Recombination analysis of four new E30 strains in this study indicated that recombination events may occur in P2, P3, and 3’-UTR regions. The recombinant donor may be E6 strain P735/CHN/2013, E18 strains (S5758/CHN/2018, S6064/CHN/2018, and S6363/CHN/2018), E3 strain 123-R2/CHN/2018, and E9 strain KM812/CHN/2010, and these possible recombinant donors have been all isolated from China. E6 strain P735/CHN/2013 was isolated from fecal samples of children with HFMD in China in 2013.The E3 strain 123-R2/CHN/2018 and 3 E18 strains, were separated in 2018 and E9 strain KM812/CHN/2010 was separated in Kunming, Yunnan Province, China in 2010. These viruses are frequently found to cocirculate among patients with HFMD, which increases the probability of recombination. The limitation of this study is that the samples collected were merely stool samples. Taking multiple samples at the same time may be helpful to the diagnosis and treatment of the disease

## Conclusion

In summary, this study extends the whole genome sequence of E30 in GenBank, in which mutations and recombinations have driven the evolution of E30 and further improved and enriched the genetic characteristics of E30, providing fundamental data for the prevention and control of diseases caused by E30. Furthermore, we demonstrated the value of continuous and extensive surveillance of enterovirus serotypes other than the major HFMD-causing viruses.

### Electronic supplementary material

Below is the link to the electronic supplementary material.

Supplementary Material 1

## Data Availability

All data generated or analyzed during this study were available from the corresponding author on reasonable request.
