# Supplementary material for: Complete genome analysis of echovirus 30 strains isolated from hand-foot-and-mouth disease in Yunnan province, China
Source: Virol J. 2023 Sep 20;20:215. doi: 10.1186/s12985-023-02179-9 (PMC10510139; doi:10.1186/s12985-023-02179-9)
Supplement: Supplementary file 1 — Supplementary Material 1 [file 12985_2023_2179_MOESM1_ESM.docx]

**Table S1.** Amplification and sequencing primers of the whole genome

| Primer | Sequence | Nucleotide position |
| --- | --- | --- |
| E201F | TTAAAACAGCCTGTGGGTTG | 1-20 |
| AN89 | CCAGCACTGACAGCAGYNGARAYNGG |  |
| AN88 | TACTGGACCACCTGGNGGNAYRWACAT |  |
| E301r | GCTCAATAGACTCTTCGC | 437-420 |
| E301f | GTGTCTAATAGAGCAATT | 643-660 |
| E302r | GCAAGCCGACGTCCCATATA | 2195-2214 |
| E303F | TGTGTGTACATCGCCCAGT | 2664-2682 |
| E303R | CGTTGAGAGCTGGTGATGAC | 2814-2833 |
| E303f  E304f  E305f  E306f  E307f  EV8R | TCTCGGGACAAAATTACC  TTCACAGAGATGACCAA  GGAGAACACAGGTGAGGT  CCCAAACATGTACATCCC  TGGATCAGTTTAGGATGA  CACCGAATGCGGAGAATTTA | 3270-3287  4092-4108  4954-4971  5717-5734  6906-6923  7400-7381 |
| E307f | TGGATCAGTTTAGGATGA | 6895-6912 |
| EV8R | CACCGAATGCGGAGAATTTA | 7381-7400 |

Note: Accession number of the reference sequence: OM677620. F is the upstream amplification primer, R is the downstream amplification primer; f is the upstream sequencing primer, r is the downstream sequencing primer; AN89 is the upstream amplification primer, AN88 is the downstream amplification primer.

**Table S2.** All positive selection sites analyzed by FUBAR model

| NO. | Site | α | β | β-α | Prob[α>β] | Prob[α<β] |
| --- | --- | --- | --- | --- | --- | --- |
| 1 | 268 | 0.569 | 2.536 | 1.967 | 0.011 | 0.972 |
| 2 | 133 | 0.643 | 2.471 | 1.828 | 0.026 | 0.938 |

**Table S3.** All positive selection sites analyzed by FEL model

| NO. | codon | α | β | α=β | *p*-value |
| --- | --- | --- | --- | --- | --- |
| 1 | 19 | 0.663 | 87.052 | 0.898 | 0.0075 |
| 2 | 117 | 0.81 | 98.424 | 1.19 | 0.0081 |
| 3 | 119 | 0.614 | 67.756 | 0.842 | 0.0086 |
| 4 | 121 | 2.737 | 396.468 | 3.792 | 0.0077 |
| 5 | 133 | 0.12 | 71.479 | 0.265 | 0.0018 |
| 6 | 145 | 1.377 | 63.025 | 1.718 | 0.0221 |
| 7 | 199 | 1.427 | 60.604 | 1.7 | 0.0217 |
| 8 | 223 | 0.473 | 433.423 | 0.684 | 0.0001 |
| 9 | 229 | 0.339 | 116.037 | 0.482 | 0.0025 |
| 10 | 233 | 2.69 | 64.884 | 3.056 | 0.0407 |
| 11 | 235 | 0.636 | 54.783 | 0.908 | 0.011 |
| 12 | 236 | 1.268 | 68.537 | 1.518 | 0.0173 |
| 13 | 239 | 0.446 | 67.879 | 0.646 | 0.0061 |
| 14 | 247 | 1.127 | 66.826 | 1.495 | 0.0169 |
| 15 | 278 | 0.501 | 68.172 | 0.723 | 0.007 |

**Table S4.** All positive selection sites analyzed by MEME model

| NO. | Site | α | β- | p- | β+ | p+ | *p*-value |
| --- | --- | --- | --- | --- | --- | --- | --- |
| 1 | 133 | 0.07 | 0.03 | 0.83 | 9.98 | 0.17 | 0 |
| 2 | 289 | 0.64 | 0 | 0.94 | 88.86 | 0.06 | 0 |
| 3 | 292 | 0.54 | 0 | 0.99 | 2047.96 | 0.01 | 0 |
| 4 | 19 | 0.51 | 0 | 0.99 | 813.18 | 0.01 | 0.01 |
| 5 | 258 | 0.56 | 0 | 0.86 | 11.01 | 0.14 | 0.01 |
| 6 | 291 | 0.64 | 0.23 | 0.99 | 2244.86 | 0.01 | 0.01 |
| 7 | 80 | 1.28 | 0.05 | 0.98 | 33.32 | 0.02 | 0.04 |
| 8 | 79 | 4.59 | 0 | 0.99 | 303.2 | 0.01 | 0.06 |
| 9 | 119 | 0.61 | 0 | 0.99 | 86.25 | 0.01 | 0.06 |
| 10 | 138 | 0.62 | 0 | 0.99 | 115.25 | 0.01 | 0.06 |
| 11 | 121 | 2.44 | 0 | 0.99 | 398.32 | 0.01 | 0.08 |
| 12 | 84 | 0.88 | 0 | 0.9 | 6.43 | 0.1 | 0.09 |
| 13 | 117 | 0.47 | 0.01 | 0.99 | 75.39 | 0.01 | 0.09 |

**Table S5.** Recombination events detected by RDP4

| Event | Recombinant | Major Parent | Minor Parent | Beginning breakpoint 99%CI | Ending breakpoint 99%CI |  |
| --- | --- | --- | --- | --- | --- | --- |
|  |  |  |  |  |  |  |
| 1 | 15K3 | KP289439_E6 | MK791150_E3 | 5655-5731 | 7020-7243 |  |
| 2 | KP289439_E6 | JN596587_E9 | 15K3 | 3802-3919 | 4857-5311 |  |
| 3 | MN815813_E18 | JN596587_E9 | KP289439_E6 | 3799-3955 | 5532-5713 |  |

**Table S6.** The P-value of the recombination event detected by different methods

| Event | Methods and P-value | | | | | | |
| --- | --- | --- | --- | --- | --- | --- | --- |
|  | RDP | GENECONV | BootScan | MaxChi | Chimaera | SiScan | 3Seq |
| 1 | 1.497X10^-33^ | 1.957X10^-11^ | 3.815X10^-41^ | 4.021X10^-24^ | 5.096X10^-26^ | 8.373X10^-26^ | 4.840X10^-14^ |
| 2 | 4.037X10^-29^ | 3.357X10^-28^ | 1.265X10^-38^ | 9.713X10^-21^ | 4.746X10^-15^ | 4.416X10^-55^ | 1.696X10^-5^ |
| 3 | 2.651X10^-35^ | 2.312X10^-27^ | 2.901X10^-44^ | 1.804X10^-13^ | 3.363X10^-26^ | 1.474X10^-27^ | 7.985X10^-15^ |
